# Bovine Herpesvirus-4 Based Vaccine Provides Protective Immunity against *Streptococcus suis* Disease in a Rabbit Model

**DOI:** 10.3390/vaccines11051004

**Published:** 2023-05-20

**Authors:** Nihua Dong, Hester Nichols, Qing Sun, Xiaojun Chen, Jiayang Zheng, Zhixin Guan, Hailong Zhang, Andrew Davison, Yvonne Wezel, Zongjie Li, Beibei Li, Ke Liu, Donghua Shao, Yafeng Qiu, Jianhe Sun, Xiangdong Li, Mathew Upton, Zhiyong Ma, Michael A. Jarvis, Jianchao Wei

**Affiliations:** 1 Shanghai Veterinary Research Institute, Chinese Academy of Agricultural Science, Shanghai 200241, China; dongnihua0519@163.com (N.D.); sun7355608520@163.com (Q.S.); chenxiaojun159753@163.com (X.C.); asd126163@163.com (J.Z.); zhixinguan2020@163.com (Z.G.); zhanghailong1997@163.com (H.Z.); lizongjie@shvri.ac.cn (Z.L.); lbb@shvri.ac.cn (B.L.); liuke@shvri.ac.cn (K.L.); shaodonghua@shvri.ac.cn (D.S.); yafengq@shvri.ac.cn (Y.Q.); zhiyongma@shvri.ac.cn (Z.M.); 2 The Vaccine Group Ltd., Derriford Research Facility, Plymouth PL6 8BX, UK; hester.nichols@thevaccinegroup.co.uk (H.N.); yvonne.wezel@thevaccinegroup.co.uk (Y.W.); 3 MRC-University of Glasgow Centre for Virus Research, Glasgow G61 1QH, UK; andrew.davison@glasgow.ac.uk; 4 Shanghai Key Laboratory of Veterinary Biotechnology, Shanghai Jiao Tong University, Shanghai 200240, China; sunjhe@sjtu.edu.cn; 5 College of Veterinary Medicine, Yangzhou University, Yangzhou 225009, China; xiaonanzhong@163.com; 6 School of Biomedical Sciences, University of Plymouth, Plymouth PL4 8AA, UK; mathew.upton@plymouth.ac.uk

**Keywords:** *Streptococcus suis*, BoHV-4, GMD, suilysin, serotype, vector-based vaccine, protection

## Abstract

*Streptococcus suis* (*S. suis*) is a bacterial pathogen of pigs that has a major animal health and economic impact on the pig industry. Bovine herpesvirus-4 (BoHV-4) is a new virus-based vaccine vector that has been used for the immunogenic delivery of antigens from a variety of pathogens. In the present study, two recombinant BoHV-4-based vectors were evaluated for their ability to induce immunity and protection against *S. suis* in a rabbit model. The GMD protein is a fusion protein consisting of multiple dominant B-cell epitopes ((B-cell dominant epitopes of GAPDH, MRP, and DLDH antigens) (BoHV-4/GMD)) and the second suilysin (SLY) (BoHV-4/SLY) from *S. suis* serotype 2 (SS2). Both GMD and SLY delivered by the BoHV-4 vectors were recognized by sera from SS2-infected rabbits. The vaccination of rabbits with the BoHV-4 vectors induced antibodies against SS2, as well as against additional *S. suis* serotypes, SS7 and SS9. However, sera from BoHV-4/GMD-vaccinated animals promoted a significant level of phagocytic activity by pulmonary alveolar macrophages (PAMs) against SS2, SS7, and SS9. In contrast, sera from rabbits immunized with BoHV-4/SLY induced PAM phagocytic activity against only SS2. In addition, BoHV-4 vaccines differed in the associated level of protection against lethal SS2 challenge, which ranged from high (71.4%) to low (12.5%) for BoHV-4/GMD and BoHV-4/SLY, respectively. These data suggest BoHV-4/GMD as a promising vaccine candidate against *S. suis* disease.

^‡^ These authors contributed equally to this work.

## 1. Introduction

*Streptococcus suis* (*S. suis*)—an encapsulated Gram-positive bacterial pathogen of pigs causing meningitis, endocarditis, and multiple other pathologies—has a major economic impact on the pig industry [1,2]. In recent years, *S. suis* has also emerged as a zoonotic pathogen, especially in eastern and Southeast Asia [3]. Based on the antigenicity of bacterial capsular polysaccharides, *S. suis* can be classified into at least 35 serotypes (ranging from serotype 1 to 34 and 1/2). The predominant serotypes in pigs are serotype 1/2, 2, 3, 7, and 9 [4,5], of which serotype 2 (SS2) is considered the dominant pathogenic serotype and is most commonly associated with zoonotic disease [6,7].

Vaccination is an attractive alternative strategy to antibiotic therapy for the control of *S. suis* infection in pigs, as it may considerably help avoid the emergence of drug-resistant strains associated with antibiotic use. Locally produced autogenous vaccines comprised of inactivated *S. suis* serotypes are available for use in the field [6]. However, these vaccines are of questionable efficacy as they appear unable to elicit cross-protection against different *S. suis* serotypes, or even between heterologous strains (sequence types, STs) of the same serotype [6,8,9]. Given that multiple serotypes with diverse sequence types are circulating in pig herds [10], a “universal” vaccine capable of providing a strong degree of cross-protection may be a promising candidate for development as a *S. suis* vaccine for the control of disease associated with this bacterium in pigs [6].

Highly conserved immunogenic proteins present across multiple *S. suis* serotypes are suitable antigenic targets for inclusion in a universal *S. suis* vaccine. In the last decade, a number of the well-conserved immunogenic proteins of *S. suis*, such as glyceraldehyde-3-phosphate dehydrogenase (GAPDH), muramidase-released protein (MRP), dihydrolipoamide dehydrogenase (DLDH), and suilysin (SLY) [11,12,13,14,15], have been identified and evaluated for their potential as universal subunit vaccine candidates [4,6]. GAPDH, MRP, and DLDH are *S. suis* virulence-associated proteins and are highly conserved in SS2, SS7, and SS9 [11,13,15]. A recombinant protein, GMD, was recently shown to provide 90% protection against SS2 challenge in mice [16]. SLY is a thiol-activated secreted hemolysin that is highly immunogenic and well-conserved among different serotypes [17,18,19]. Passive immunization with antisera against SLYP353L (a recombinant non-hemolytic SLY mutant with a Pro-to-Leu point mutation at amino acid position 353) was shown to protect mice from acute death following *S. suis* challenge [17]. This finding translated to pigs, with animals immunized with purified SLY exhibiting only mild clinical signs following *S. suis* challenge that subsided more rapidly than those receiving a placebo control [12]. Together, these observations suggest that GMD and SLY represent promising targets for the development of a universal subunit vaccine against *S. suis*.

Bovine herpesvirus-4 (BoHV-4) is a double-stranded (ds) DNA virus belonging to the gamma herpesvirinae sub-family [20]. BoHV-4 is non-oncogenic and infection is not directly linked to any specific pathology in either its natural cattle host nor in multiple other ruminant species where the virus appears to occur naturally [21]. Consistent with wide host tropism, BoHV-4 is capable of replicating in vitro in cells from a variety of animal species [21]. BoHV-4 has also been shown to be able to experimentally infect a variety of animal species in addition to ruminants, including rabbits [22,23] and pigs [24,25]. BoHV-4 has been shown to be highly immunogenic in these species. This high immunogenicity, combined with the lack of pathogenicity and capacity for accommodating large amounts of foreign genetic material [23], has resulted in considering BoHV-4 as a promising vaccine vector. To date, BoHV-4 vectors have been used to deliver a variety of heterologous antigens from different pathogens in several animal models [21,23,25,26,27,28,29].

In the current study, we constructed recombinant BoHV-4 vectors expressing GMD and SLY, antigens that had previously been identified as promising targets for inclusion in a universal subunit vaccine for *S. suis* [12,16,17]. Immunity and protective efficacy against *S. suis* induced by the corresponding BoHV-4/GMD and BoHV-4/SLY vaccines were then evaluated in a rabbit *S. suis* disease model.

## 2. Materials and Methods

### 2.1. Ethics Statement

All animal experiments were approved by the Institutional Animal Care and Use Committee of Shanghai Veterinary Research Institute, China (IACUC No: SVLAC/XM-M-22002) and performed in compliance with the Guidelines on the Humane Treatment of Laboratory Animals (Ministry of Science and Technology of the People’s Republic of China, Policy No. 2006 398).

### 2.2. Virus, Cells, and S. suis Strains

The BoHV-4 V. test strain (Genbank No JN133502) used in this study was initially isolated from cattle [30] and was subsequently cloned as an infectious bacterial artificial chromosome (BAC) [31]. MadinDarby bovine kidney (MDBK; ATCC CCL-22) cells were cultured in minimum essential media (MEM) containing 10% fetal bovine serum (FBS), 1% penicillin–streptomycin–glutamine and 1% non-essential amino acids. Cells were incubated at 37 °C in a normal atmosphere supplemented with 5% CO_2_ and used for the reconstitution of recombinant BoHV-4/GMD and BoHV-4/SLY BACs and preparation of virus stocks. SS2 (ZY05719 strain), SS7 (SH04815 strain), and SS9 (SH26 strain) were in-house field isolates previously recovered from diseased pigs in China [32]. All *S. suis* isolates were grown in tryptone soya broth (OXOID, Basingstoke, UK) supplemented with 5% fresh sheep blood under static condition at 37 °C. To count SS2, first aspirate 100 μL of SS2 bacterial solution, dilute into a pre-prepared 900 μL sterile PBS solution, dilute to 10^−6^ at a 10-fold ratio, apply 100 μL dilution on Colombian blood agar plates, replicate 4 times per dilution, invert in a 37 °C constant temperature incubator, and record the number of colonies on the plates the next day.

### 2.3. Construction of Recombinant BoHV-4/GMD and BoHV-4/SLY BACs Expressing S. suis Antigens

**Figure 1:**
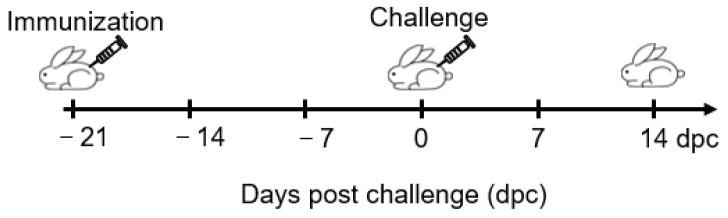
Schematic diagram of immunization and challenge.

The strategy for constructing recombinant BoHV-4/GMD and BoHV-4/SLY BACs expressing *S. suis* antigens is shown in Figure 1. A wild-type (WT) BAC of BoHV-4 (BoHV-4/WT BAC) generated from a BoHV-4 V. test strain [31] was re-engineered to contain a Cre recombinase within the BAC cassette [33]. This resulted in the creation of a recombinant BAC (BoHV-4/WTCRE) with the capacity for BAC cassette self-excision following introduction into MDBK cells during virus reconstitution. Genes encoding GMD [16] and SLY (GenBank No. KC405589.1) *S. suis* proteins were codon-optimized for expression in swine (*Sus scrofa*) and chemically synthesized (GeneArt, Thermo Fisher Scientific, UK) as plasmids and grown in Top10 *E. coli* (Invitrogen™, Thermo Fisher Scientific, UK), cultured in LB broth with carbicillin (100 μg/mL final concentration). The synthetic genes were ligated into a transfer plasmid (pMiniOri), which contained an expression cassette consisting of the human CMV promoter (hCMV), R6K origin of replication (R6K), and a kanamycin resistance marker (KanR) flanked by FLP recombinase recognition target (FRT) sites (Figure 1). The transfer plasmid also contained homology regions (HRs) flanking the ORF73 gene of BoHV-4 required for insertion of GMD and SLY expression cassettes within the BoHV-4/WTCRE BAC. The recombinant transfer plasmids were grown in PirI *E. coli* (Invitrogen™, Thermo Fisher Scientific, UK) cultured in LB broth with kanamycin (50 μg/mL final concentration). Following linearization of the transfer plasmids by restriction enzyme digestion, GMD or SLY expression cassettes were recombined into the BoHV-4/WTCRE BAC to replace the ORF73 gene by using homologous recombination [34] in EL250 *E. coli*, cultured in LB broth with chloramphenicol and kanamycin (17.5 μg/mL and 25 μg/mL final concentration, respectively). This resulted in the creation of recombinant BoHV-4 BAC clones BoHV-4/GMD and BoHV-4/SLY. The FRT-flanked KanR cassette was removed from BoHV-4/GMD and BoHV-4/SLY BACs by arabinose-induced expression of FLP recombinase [35]. Recombinant BACs were initially screened by restriction fragment length polymorphism (RFLP) analysis following digestion of BAC DNA with selected individual restriction enzymes (SpeI, XbaI, BamHI, XmaI, HindIII) depending on predicted band patterns. A single enzyme was used prior to KanR removal and BACs were confirmed by digestion using three separate enzymes post KanR removal (Figure S1). Expected digest patterns were predicted by MacVector™ Inc software (Cambridge, UK) in comparison to the WT BoHV-4 BAC. After digestion for four hours at 37 °C, samples were separated on a 1% agarose gel electrophoresis, and visualized under UV light.

### 2.4. Genomic Characterization of BoHV-4 BACs

Correct insertion of *S. suis* antigen expression cassettes within BoHV-4/GMD and BoHV-4/SLY BACs to replace ORF73 was confirmed by PCR using primers that bound either side of the ORF73 gene of BoHV-4 (ORF73 outside forward primer: 5′-ACACAACCCCACAACCCATT-3′ and ORF73 outside reverse primer: 5′-AGTTGCTGCTCTGGTCTTCC-3′). PCR was performed using AccuPrime™ Taq DNA Polymerase System (Invitrogen™) and the expected PCR sizes were 4900 bp for SIY, 4519 bp for GMD, and 3015 bp for the WT containing ORF73. The purified PCR products were sequenced by direct DNA Sanger sequencing (Mix2Seq kit, Eurofins Genomics, Ebersberg, Germany). Whole genome next generation sequencing (NGS) using the Illumina platform (Novogene, Cambridge, UK or Andrew Davison, MRC-University of Glasgow Centre for Virus Research, Glasgow, UK) was used to confirm complete BAC genome fidelity. Sequencing results were analyzed using MacVector, Inc software (Cambridge, UK) by alignment to a reference based on the BoHV-4/WT BAC sequence.

### 2.5. Reconstitution of Recombinant BoHV-4/GMD and BoHV-4/SLY Viruses

BoHV-4/GMD and BoHV-4/SLY viruses were reconstituted by transfection into MDBK cells using PolyJet™ DNA In Vitro Transfection Reagent (SignaGen, Rockville, MD, USA) according to the manufacturer’s recommendations, with an additional 30 min centrifugation step at 310× *g* prior to incubation. Cells were then cultured at 37 °C with 5% CO_2_ and monitored for cytopathic effect (CPE) for 14 days. Supernatant from cells with full CPE was collected and stored at −80 °C. Reconstituted viruses were then passaged in MDBK cells for five passages, and expression of V5-tagged GMD and SLY were confirmed at each passage by Western immunoblot. DNA encoding GMD and SLY was amplified by PCR from passage 5 (p5) viruses and sequenced to confirm presence of *S. suis* target genes. Complete genome integrity of p5 viruses was confirmed by NGS. Virus titers were measured in MDBK cells using a 50% tissue culture infective dose (TCID50) assay [36].

### 2.6. Western Immunoblot Analysis of S. suis Antigen Expression

Protein cell extracts were obtained from 25 cm^2^ confluent flasks of MDBK cells infected with BoHV-4/GMD, BoHV-4/SLY, or BoHV-4/WT by adding 100 μL of cell extraction buffer (50 mM Tris–HCl, 150 mM NaCl, and 1% NP-40; pH 8). To analyze cell extracts, a 10% SDS-PAGE gel electrophoresis was used. After protein transfer in PVDF membranes by electroblotting, the membranes were incubated with either convalescent rabbit sera specific to SS2 or anti-V5 antibodies (Bio-Rad, Hercules, CA, USA) as described previously [37]. Then, they were probed with horseradish peroxidase-labeled anti-rabbit immunoglobulin (SIGMA), diluted 1:10,000.

### 2.7. Preparation of Recombinant BoHV-4 Virus Stocks

Supernatants of reconstituted p2 BoHV-4/GMD and BoHV-4/SLY viruses were used for preparation of virus stocks. Briefly, MDBK cells pre-cultured to 80–90% confluency were inoculated with p2 virus supernatants, followed by culture at 37 °C with 5% CO_2_ for 3–5 days. Cells were harvested just prior to 100% CPE by scraping cells into the media followed by a single cycle of rapid freeze–thaw. Supernatants were clarified by centrifugation at 3100× *g* for 20 min at room temperature, and then layered onto a 20% sorbitol cushion followed by ultra-centrifugation at 70,000× *g* for 80 min at room temperature. Virus pellets were resuspended in Dulbecco’s modified Eagle’s medium (DMEM), centrifuged at 310× *g* for 5 min at room temperature, and then aliquoted and stored at −80 °C. Virus titers were measured in MDBK cells using a TCID50 assay, and expression of V5-tagged GMD and SLY was confirmed by Western immunoblot with anti-V5 antibodies and rabbit sera specific to SS2. Absence of bacterial contamination was confirmed by the lack of bacterial growth in viral stocks inoculated into LB broth and cultured at 37 °C for 2 days in the absence of antibiotics. NGS of DNA prepared from virus stocks was used to confirm genome integrity of all viruses.

### 2.8. Rabbit Immunization and S. suis Challenge

Two-month-old New Zealand rabbits were purchased from Shanghai JieSiJie Laboratory Animal Co., Ltd. Shanghai, China, and confirmed as negative for *S. suis* antibodies using an enzyme-linked immunosorbent assay (ELISA). Previously, antibody responses induced by BoHV-4 based vaccine vectors have been evaluated in multiple animal species via different routes of administration, including intramuscular (IM) [25]; intravenous (IV) [23]; intraperitoneal (IP) [21]; and intranasal (IN) [29]. To test the antibody response following vaccination with different routes, nine rabbits were divided into three groups of three rabbits each and vaccinated with BoHV-4/GMD, BoHV-4/SLY, and BoHV-4 WT at a dose of 1.52 × 10^9^ TCID50 via intramuscular (IM), subcutaneous (SC), or intranasal (IN) routes, as indicated.

To test immune correlation, the rabbits were randomly divided into experimental (immunized) and DMEM (diluent control) groups. We divided 24 rabbits into three groups of nine rabbits each, and vaccinated them with BoHV-4/GMD, BoHV-4/SLY and DMEM at a dose of 1.52 × 10^9^ TCID50 via intramuscular (IM) injection. Rabbits were monitored for 21 days. Blood samples were collected at 0, 7, 14, 21 dpi for analysis of antibody responses by ELISA. Rabbits are susceptible to SS2 infection and the pathology of rabbits experimentally infected with SS2 via the IP route is similar to that observed in pigs [38]. Immune-associated rabbits were challenged intraperitoneally with 5.96 × 10^6^ colony forming units (CFUs) of SS2 ZY05719 strain at 21 days post-immunization (dpi) and monitored at least daily over 14 days for symptomology and scored accordingly as shown in Figure 1. The rectal temperature of each animal was measured at −21, −14, −7, 0, 2, 4, 7, 9, 11, 12, and 14 dpc. The rabbits exhibited extreme lethargy and were regarded as terminally moribund, and humanely euthanized in accordance with the Guidelines on the Humane Treatment of Laboratory Animals (Ministry of Science and Technology of the People’s Republic of China, Policy No. 2006 398).

### 2.9. ELISA for Detection of S. suis Antibodies

Blood samples were collected from rabbits at times indicated for analysis of antibodies against *S. suis* bacteria and recombinant GMD by ELISA. The optimal working concentration of GMD coating was s 0.84 μg/mL. For a source of bacterial antigen, SS2, SS7, and SS9 were inactivated by formaldehyde and coated onto ELISA plates at 5 × 10^5^ CFU per well. Plates were then incubated at 4 °C overnight and blocked with 5% bovine serum albumin. The experimental rabbit sera were diluted to 1:200 before adding to the antigen-coated plates. Antibody binding was visualized by incubation with horseradish peroxidase-conjugated goat anti-rabbit IgG (Thermo Fisher Scientific, Shanghai, China) using 3,3′,5,5′-tetramethylbenzidine as the substrate. Detection of antibody against GMD by ELISA was performed in a similar fashion, and as described previously [39]. Level of antibody binding was quantified based on optical density at 450 nm (OD450). The cutoff value for a negative well was calculated as three times the standard deviation plus the average OD450 of the control group.

### 2.10. ELISA for Detection of IL-4 and IFN-γ

Blood samples were collected from the immunized rabbits at 0, 7, and 14 dpi followed by analysis of IFN-γ and IL-4 levels by ELISA using commercially available kits (Cusabio, Houston, TX, USA). First diluting the standard, setting the standard wells and sample wells, operating left and right as directed, and finally using a microplate reader set to 450 nm, the optical density of each well was determined in 5 min. All analyses were performed according to the manufacturer’s instructions.

### 2.11. Antibody-Mediated Phagocytic Activity against S. suis

Antibody-mediated phagocytic activity against *S. suis* in rabbits from 21 dpi was measured in pulmonary alveolar macrophages (PAMs), as described previously [40]. PAMs were prepared from *S. suis* naïve 3-month-old New Zealand rabbits (JieSiJie Laboratory Animal Co., Ltd.), as described previously. Rabbits were first aseptically dissected to obtain intact lung tissue, the lungs were immediately placed in pre-chilled PBS solution, and rabbit alveolar macrophages were subsequently isolated by bronchial lavage [41,42]. Briefly, SS2, SS7, and SS9 cultured to an OD600 of 0.6 were inactivated by incubation at 121 °C for 15 min, followed by surface labeling with fluorescein isothiocyanate (FITC), as described previously [43]. FITC-labeled bacteria (10^7^ cells) were then incubated with sera from the 21 dpi rabbit sera (5 μL) for 45 min at 37 °C to allow for opsonization. Opsonized bacteria were subsequently incubated with rabbit PAMs at a multiplicity of infection (moi) of 300 bacteria per cell for 2 h at 37 °C. Non-phagocytosed bacteria were removed with a PBS wash and residual extracellular fluorescence was quenched with trypan blue. Fluorescence intensities representing phagocytized bacteria were measured with a microplate fluorometer at 485 nm excitation/535 nm emission (M3; Molecular Devices, Sunnyvale, CA, USA). Phagocytotic activity was calculated as follows: percentage phagocytosis (% of DMEM control) = (fluorescence intensity of immunized rabbits/fluorescence intensity of DMEM control rabbits) × 100.

### 2.12. Statistical Analysis

Values presented in this study are provided as mean ± standard deviation. Significance was determined using Student’s *t*-test or two-way ANOVA. A value of *p* < 0.05 was considered statistically significant.

## 3. Results

### 3.1. Construction of BoHV-4/GMD and BoHV-4/SLY BACs Expressing S. suis Antigens

**Figure 2:**
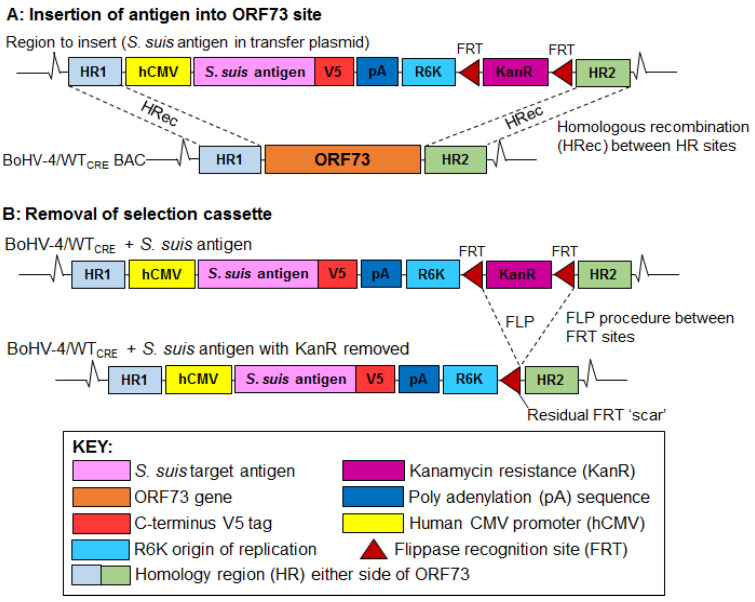
Construction of BoHV-4/GMD and BoHV-4/SLY BACs expressing *S. suis* antigens. Genes encoding GMD and SLY were ligated into a transfer plasmid that contains hCMV, R6K, KanR, as well as homology regions (HRs) flanking the ORF73 gene of BoHV-4. The resulting GMD or SLY expression cassettes were recombined into BoHV-4/WTCRE BAC genome by homologous recombination to replace the ORF73 gene to generate recombinant BoHV-4/GMD and BoHV-4/SLY BACs. Following KanR removal by FLP recombinase induction, BoHV-4/GMD and BoHV-4/SLY viruses were reconstituted in MDBK cells.

To construct BoHV-4/GMD and BoHV-4/SLY BACs expressing *S. suis* antigens, GMD or SLY expression cassettes were constructed by inserting the synthetized GMD and SLY gene segments into the transfer plasmid containing the hCMV promoter, R6K origin of replication, and KanR (Figure 2). GMD and SLY expression cassettes were analyzed through restriction digest using NheI and SphI. The resulting GMD or SLY expression cassettes were recombined into the BoHV-4/WTCRE BAC genome. The GMD and SLY expression cassettes were inserted within the BoHV-4 genome to replace ORF73—a gene dispensable for virus replication in vitro, but essential for virus persistence in vivo [44]. Following confirmation of BACs through RLFP as above, removal of the KanR marker from BoHV-4/GMD and BoHV-4/SLY BACs was performed using the FLP recombinase (flippase) method [35], and confirmed by RLFP using three separate restriction enzymes (Figure S1). PCR and sequencing were performed to establish correct construction of the expected BAC. The respective recombinant viruses were reconstituted in MDBK cells during which the BAC cassettes were removed concomitant with expression of the Cre recombinase located within the BAC cassette.

### 3.2. Reconstitution and Characterization of BoHV-4/GMD and BoHV-4/SLY

**Figure 3:**
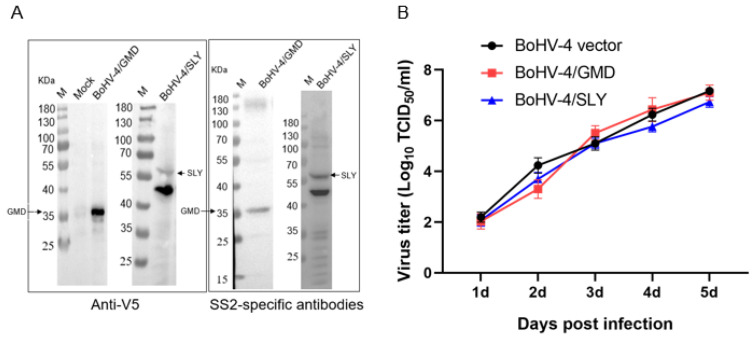
Reconstitution and characterization of BoHV-4/GMD and BoHV-4/SLY. (**A**) Analysis of GMD and SLY expression by Western immunoblot. BoHV-4/GMD and BoHV-4/SLY virus stocks were prepared from p2 viruses on MDBK cells and expression of GMD and SLY was confirmed by Western immunoblot with anti-V5 antibody and rabbit sera specific to SS2. (**B**) Replication kinetics of BoHV-4/GMD and BoHV-4/SLY. The replication kinetics of BoHV-4/GMD and BoHV-4/SLY were determined in MDBK cells using a TCID50 assay and compared with parental WT BoHV-4.

Following the reconstitution of BoHV-4/GMD and BoHV-4/SLY vaccine vectors in MDBK cells, the expression of the *S. suis* target antigen was analyzed by Western immunoblot. Stability of *S. suis* transgene expression was demonstrated over multiple (five) passages of the BoHV-4 *S. suis* vectors in MDBK cells (Figure S2) based on V5 epitope tag positivity. Reconstituted BoHV-4/GMD and BoHV-4/SLY viruses collected from p2 were used for preparation of virus stocks. In addition to V5 reactivity, GMD and SLY were also shown to be reactive with convalescent rabbit sera specific to SS2, indicating that both GMD and SLY antigens delivered by the BoHV-4 vector were being expressed in a form that was recognizable by SS2-specific antibodies induced during normal SS2 infection (Figure 3A). Growth characteristics of BoHV-4/GMD and BoHV-4/SLY stock viruses were compared to parental WT BoHV-4 and were shown to be comparable (Figure 3B), indicating that the insertion of GMD and SLY had no significant effect on replication. Together, these data show that BoHV-4/GMD and BoHV-4/SLY delivering GMD and SLY stably expressed their *S. suis* transgene in a form that was recognizable during infection with the live form of the *S. suis* bacterium, and that the presence of the transgene had no notable effect on the in vitro replication of the vaccine vector.

### 3.3. Antibody Responses in Rabbits Immunized with BoHV-4/GMD via Different Administration Routes

**Figure 4:**
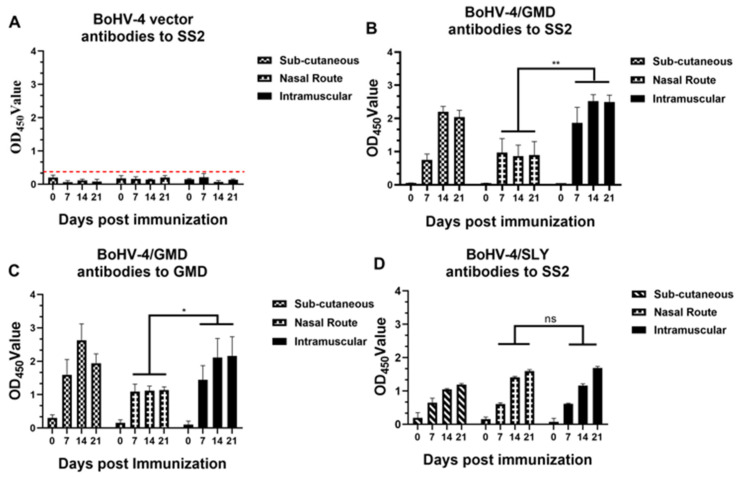
Analysis of antibody response in rabbits immunized BoHV-4/GMD via different administration routes. Rabbits (*n* = 3) were immunized with BoHV-4/GMD or BoHV-4 WT viruses via IM, SC, and IN routes. Blood samples were collected at 0, 7, 14, and 21 dpi for detection of antibodies against SS2 and GMD. (**A**) Antibody response against SS2 in rabbits immunized with BoHV-4 vector. (**B**) Antibody response against SS2 in rabbits immunized with BoHV-4/GMD. (**C**) Antibody response against GMD in rabbits immunized with BoHV-4/GMD. (**D**) Antibody response against SS2 in rabbits immunized with BoHV-4/SLY. Red dotted line indicates the cut-off value for negative. *, *p* < 0.05; **, *p* < 0.01 examined by paired Student’s *t*-test between groups. ns, no significant difference between groups tested by paired Student’s *t*-test.

To identify the administration route that induced the highest antibody response in our studies, blood samples were collected from the immunized rabbits at 0, 7, 14, and 21 dpi for analysis of antibody responses by ELISA. As shown in Figure 4A, antibodies against SS2 were not detected in rabbits receiving the WT BoHV-4 vector by any route, indicating that all detected responses were due to SS2 reactivity induced by the *S. suis* antigen encoded by the BoHV-4 vector. SS2-specific antibody responses were observed in rabbits immunized with BoHV-4/GMD and BoHV-4/SLY (Figure 4B,D), becoming detectable in all groups from 7 dpi. Comparison of antibody levels in animals vaccinated with BoHV-4/GMD via different routes showed that the levels in rabbits immunized with IM were relatively higher than those immunized with SC and significantly higher than those immunized via the IN route (Figure 4B). However, there was no significant difference in antibody levels between the three routes in the BoHV-4/SLY-vaccinated group (Figure 4D). Antibodies against the recombinant GMD protein were also analyzed by ELISA. GMD antibody responses paralleled those against SS2, with the lowest levels again being observed in rabbits immunized via the IN route. These data identify the IM route as the most efficacious for induction of SS2/GMD antibodies by BoHV-4/GMD in rabbits. Therefore, combining the results of antibody levels after immunization and considering that the IM route is commonly used for on-site vaccination, the IM route was chosen for evaluation of BoHV-4/GMD and BoHV-4/SLY in all subsequent experiments.

### 3.4. BoHV-4/GMD and BoHV-4/SLY Induce Antibody Responses in Rabbits against SS2, SS7, and SS9

**Figure 5:**
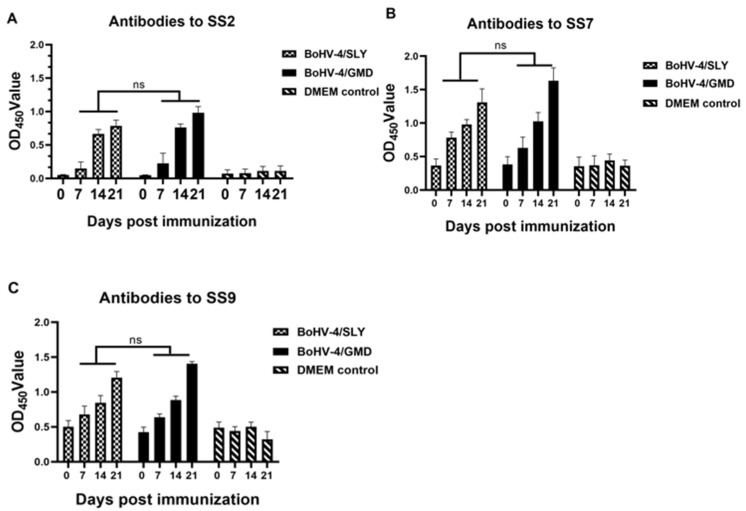
Analysis of antibody response specific to SS2, SS7, and SS9 in immunized rabbits. Rabbits (*n* = 8) were immunized with BoHV-4/SLY via IM route. One rabbit in the BoHV-4/GMD group (*n* = 7) died due to human error in blood collection. Blood samples were collected at 0, 7, 14, and 21 dpi for detection of antibodies against SS2, SS7, and SS9 by ELISA. (**A**) Levels of antibody against SS2. (**B**) Levels of antibody against SS7. (**C**) Levels of antibody against SS9. Red dotted line indicates the cut-off value for negative. ns, no significant difference between groups tested by paired Student’s *t*-test.

Antibody responses specific to SS2, SS7, and SS9 were examined using the corresponding inactivated bacteria as the coating antigens. As shown in Figure 5A–C, with the exception of a delay in antibodies against SS2 in BoHV-4/SLY-immunized rabbits, antibodies against SS7 and SS9 were detectable in rabbits immunized with BoHV-4/GMD and BoHV-4/SLY by 7 dpi. Responses then increased in all BoHV-4/GMD- and BoHV-4/SLY-immunized animals from 14 to 21 dpi, with the highest antibody levels being observed at 21 dpi for all three *S. suis* serotypes. No significant differences in antibody levels were observed between BoHV-4/GMD and BoHV-4/SLY groups. These data showed the that immunization of rabbits with BoHV-4/GMD and BoHV-4/SLY produced antibodies against SS2 that were cross-reactive against SS7 and SS9 *S. suis* serotypes.

### 3.5. Levels of Cytokines in Rabbits Immunized with BoHV-4/GMD and BoHV-4/SLY

**Figure 6:**
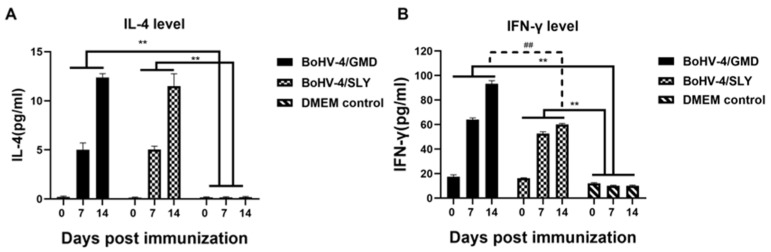
Levels of IL-4 and IFN-γ in immunized rabbits. Rabbits were immunized with BoHV-4/GMD (*n* = 7) or BoHV-4/SLY (*n* = 8) via IM route. Blood samples were collected at 0, 7, and 14 dpi post immunization for detection of IL-4 and IFN-γ production by ELISA. (**A**) Levels of IL-4 production. (**B**) Levels of IFN-γ production. **, *p* < 0.01 tested by the two-way ANOVA between groups. ##, *p* < 0.01 tested by tested by Student’s *t*-test between groups.

IFN-γ and IL-4 are used as indicators of Th1- and Th2-type immune responses, respectively [43,45,46,47], with both types of response being thought to be important for *S. suis* control [6]. We therefore analyzed IFN-γ and IL-4 production in the rabbits immunized with BoHV-4/GMD and BoHV-4/SLY vaccines. Both BoHV-4/GMD and BoHV-4/SLY groups displayed a significantly higher level of IFN-γ and IL-4 production compared to DMEM controls (Figure 6). The levels of IL-4 were similar between rabbits receiving BoHV-4/GMD and BoHV-4/SLY (Figure 6A) at all time points, but IFN-γ levels increased to significantly higher levels in BoHV-4/GMD- compared to BoHV-4/SLY-immunized animals by 14 dpi (Figure 6B).

### 3.6. Protective Efficacy of BoHV-4/GMD and BoHV-4/SLY against SS2 Challenge in Rabbits

**Figure 7:**
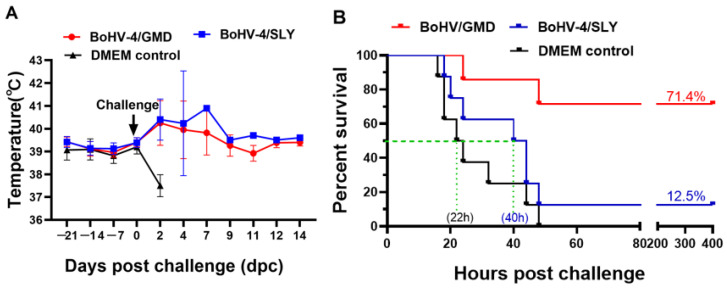
Analysis of protective efficacy of BoHV-4/GMD and BoHV-4/SLY against SS2 challenge in rabbits. Rabbits were immunized with BoHV-4/GMD (*n* = 7) or BoHV-4/SLY (*n* = 8) via IM route and challenged at 21 days post-immunization. The rabbits were monitored daily for an additional 14 days. (**A**) Changes in rectal temperature. (**B**) Survival analysis of rabbits after challenge.

Rabbits are susceptible to SS2 infection and the pathology of rabbits experimentally infected with SS2 via the IP route is similar to that observed in pigs [38]. We therefore used the SS2 rabbit model to evaluate the protection elicited by BoHV-4/GMD and BoHV-4/SLY against SS2 infection. Rabbits were immunized IM with BoHV-4/GMD and BoHV-4/SLY as shown in Figure 1. Over the 21-day vaccination phase, no experimental animals displayed any clinical symptomology associated with the vaccination, with all experimental and control animals exhibiting normal temperature values over this time period, with a range of 38.7 ± 0.7 to 39.7 ± 0.6 °C (Figure 7A). All eight rabbits in the DMEM control group rapidly developed clinical signs and were humanely euthanized within 48 h, with median survival time of 22 h after SS2 challenge. In contrast, five rabbits in the group immunized with BoHV-4/GMD survived, representing a survival of 71.4% (5/7) (Figure 7B). These surviving BoHV-4/GMD rabbits exhibited mild clinical signs, including lethargy, ruffled hair, and sustained pyrexia with rectal temperature of approximately 40 °C for 7 days (Figure 7A). Seven rabbits in the group immunized with BoHV-4/SLY succumbed to disease with a median survival time of 40 h, representing a survival of 12.5% (1/8). Overall, vaccination with BoHV-4/GMD, but not BoHV-4/SLY, provided a high level of protection against SS2 challenge in rabbits compared to either BoHV-4/SLY or DMEM controls.

### 3.7. Antibody-Mediated Phagocytic Activity against SS2, SS7, and SS9 in Immunized Rabbits

**Figure 8:**
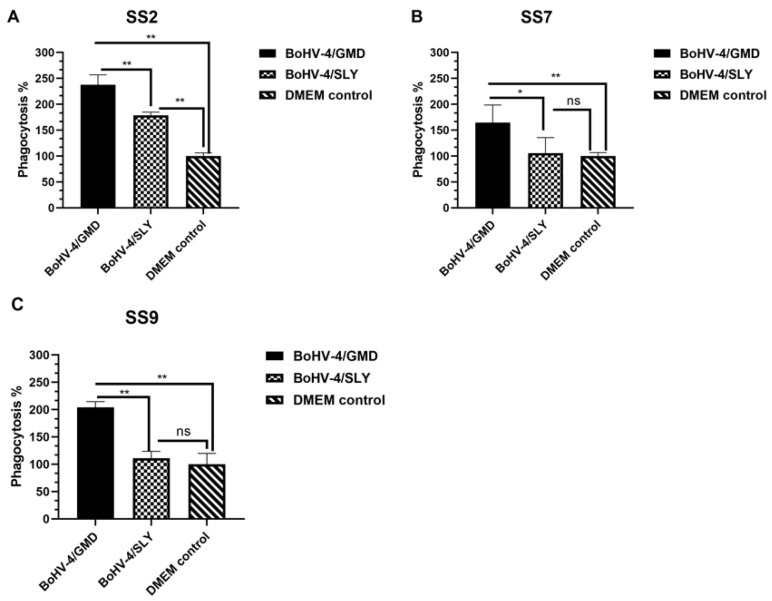
Analysis of antibody-mediated phagocytic activity against SS2, SS7, and SS9. Sera were collected from rabbits immunized with BoHV-4/GMD (*n* = 8) or BoHV-4/SLY (*n* = 8) at 21 dpi and incubated with FITC-labeled SS2, SS7, and SS9, respectively. The opsonized bacteria were subsequently incubated with PAMs from naïve rabbits. Fluorescence intensities representing the phagocytized bacteria were measured at 485 nm excitation/535 nm emission. Phagocytotic activity was calculated as follows: percentage phagocytosis (% of DMEM control) = (fluorescence intensity of immunized rabbits/fluorescence intensity of DMEM control rabbits) × 100. (**A**) Phagocytic activity against SS2. (**B**) Phagocytic activity against SS7. (**C**) Phagocytic activity against SS9. Red dotted line indicates the cut off value for negative. ns, no significant difference between groups tested by the paired Student’s *t*-test. *, *p* < 0.05; **, *p* < 0.01 tested by Student’s *t*-test between groups.

Rabbits are not susceptible to SS7 and SS9 infection, and therefore we were unable to evaluate the protective efficacy of BoHV-4/GMD and BoHV-4/SLY against these serotypes in rabbits. However, given that opsonin-dependent phagocytosis represents a primary defense mechanism against *S. suis* in vivo [6], we sought to determine whether sera from BoHV-4/GMD- and BoHV-4/SLY-immunized rabbits, which had antibodies against all three serotypes (see Figure 5), were able to promote the phagocytic activity of PAMs against SS2, SS7, and SS9. FITC-labeled *S. suis* bacteria were opsonized with sera from rabbits immunized with BoHV-4/GMD and BoHV-4/SLY followed by incubation with naïve rabbit PAMs. PAM phagocytic activity, indicated as percentage phagocytosis, was then compared between groups. In comparison to DMEM controls, sera from rabbits immunized with BoHV-4/GMD promoted significantly higher levels of phagocytic activity against all three serotypes: SS2, SS7, and SS9. In contrast, sera from rabbits immunized with BoHV-4/SLY had increased phagocytic activity against only SS2 (Figure 8A), but not SS7 (Figure 8B) nor SS9 (Figure 8C). Comparison of phagocytic activity between BoHV-4/GMD and BoHV-4/SLY groups showed that phagocytic activity against all three serotypes, including SS2, were significantly higher in the BoHV-4/GMD than the BoHV-4/SLY group (Figure 8). These results suggest that sera from rabbits immunized with BoHV-4/GMD opsonized SS2, SS7, and SS9 more efficiently, and were able to induce phagocytic activity against these three serotypes to higher levels compared to sera from BoHV-4/SLY animals.

## 4. Discussion

BoHV-4 has been identified as a vaccine vector that is able to deliver antigens from different pathogens in multiple divergent animal species, including rabbits [22,23] and pigs [24,25]. Given the relative high cost as well as ethical concerns of testing a new prototype vaccine in pigs, a rabbit *S. suis* immunogenicity/challenge model was used in this study for the initial evaluation of immune response and protection efficacy of BoHV-4/GMD and BoHV-4/SLY against *S. suis* infection. The ability to translate findings from this model into pigs is supported by the observation that the pathology of rabbits experimentally infected with SS2 via the IP route is similar to that observed in pigs [38].

*E. coli* expressed recombinant GMD [16] and SLY [12,19] had been used to identify these antigens as promising targets for development of subunit vaccines against *S. suis*. We therefore examined the antigenicity of GMD and SLY delivered by BoHV-4/GMD and BoHV-4/SLY by Western immunoblot and observed that the BoHV-4-expressed GMD and SLY were expressed in a form that was recognized by SS2-specific sera obtained from convalescent rabbits experimentally infected with SS2. The immunization of rabbits with BoHV-4/GMD and BoHV-4/SLY was then shown capable of inducing specific antibodies that bound to SS2, SS7, and SS9 and promoted the phagocytic activities of macrophages against *S. suis*. Together, these results show that both GMD and SLY, when expressed by the BoHV-4 vector, were able to elicit a *S. suis*-specific antibody response in animals. Th1- and Th2-type immunity are believed to contribute to resistance against *S. suis* infection [46,47]. High levels of IFN-γ and IL-4 as markers of Th1- and Th2-type immunity, respectively, were observed in rabbits immunized with BoHV-4/GMD and BoHV-4/SLY (compared DMEM controls). This suggests that both BoHV-4/GMD and BoHV-4/SLY were able to induce Th1- and Th2-type immune responses, which may be important for the control of *S. suis* infection in these animals.

Given that antibody responses specific to SS2, SS7, and SS9 and Th1- and Th2-type associated cytokines were observed in rabbits immunized with BoHV-4/GMD and BoHV-4/SLY, we challenged the immunized rabbits with SS2 to assess their ability to control *S. suis* infection. In addition to being the only serotype for which there is an available rabbit model, SS2 is also the most virulent and prevalent serotype in pigs and humans worldwide [6]. Remarkably, survival analysis showed that a single vaccination using BoHV-4/GMD conferred 71.4% protection against SS2 challenge; BoHV-4/SLY, in contrast, provided only 12.5% protection against SS2 challenge. Previous studies in mouse SS2 challenge models have shown a protection level of 90% and 60% for *E. coli*-expressed GMD and SLY, respectively [16,19]. These differences between the efficacy of the BoHV-4 vaccines in rabbits in our study and *E. coli*-expressed proteins in the two earlier mouse studies may be due to multiple factors beyond vaccine modality, including stringency of disease in the two species, differences in SS2 strains, and numbers of vaccine doses. However, our present findings are in good agreement with this earlier work in that they suggest the superiority of GMD in terms of immunogenicity and protection [16], and support the value of evaluating GMD-containing vaccines in pigs in future studies.

SS7 and SS9 are also predominant serotypes seen during pig infection [48]. However, we were unable to evaluate the protective efficacy of BoHV-4/GMD and BoHV-4/SLY against SS7 and SS9 in vivo, as rabbits are not susceptible to these serotypes. Although we observed specific antibody responses against SS2, SS7, and SS9 in the immunized rabbits, the binding of antibodies may not accurately reflect the level of protection against these different serotypes being induced by vaccination [12,16,19]. Opsonin-dependent phagocytosis is believed to represent a primary defense mechanism against *S. suis* in vivo [6]. We therefore analyzed antibody-mediated phagocytic activity against all three serotypes in the immunized rabbits as a surrogate biological measure to estimate the protective efficacy of BoHV-4/GMD and BoHV-4/SLY against SS7 and SS9 (in addition to SS2). Sera from rabbits immunized with BoHV-4/GMD promoted high levels of phagocytic activity in macrophages not only against SS2, but also against SS7 and SS9. Extrapolating from the survival data in the SS2 challenge experiment, this finding suggests that BoHV-4/GMD may also be able to provide a level of cross-protection against SS7 and SS9 challenge. Sera from rabbits immunized with BoHV-4/SLY induced detectable but, notably, significantly lower levels of phagocytic activity against SS2 compared to BoHV-4/GMD, which is consistent with the lowered survival rate of animals immunized with this vector compared to BoHV-4/GMD. Importantly, no increased activity was observed against SS7 and SS9, suggesting that BoHV-4/SLY may not protect the immunized animals from SS7 and SS9 infection. However, such speculations will need to await validation in an SS7 and SS9 efficacy model in future work.

BoHV-4/GMD was superior to BoHV-4/SLY at the multiple levels measured, including protective efficacy against SS2; antibody-mediated phagocytic activity against SS2, SS7, and SS9; as well as the level of IFN-γ induction. The levels of antibodies of SS2, SS7, and SS9 were similar between BoHV-4/GMD and BoHV-4/SLY. However, the antibody responses induced by SLY were not accompanied by increased antibody-mediated phagocytic activity and protective efficacy. These results are in agreement with the previous observation according to which immunization with an attenuated *S. suis* serotype 2 strain elicited SLY-specific neutralization titers, but failed to induce sufficient opsonizing antibody titers and protective immunity against SS2 and SS9 challenges in piglets [49]. GMD is an artificially constructed antigen consisting of three epitopes of GAPDH, eight epitopes of MRP, and four epitopes of DLDH, which is being developed as a universal subunit vaccine against *S. suis* infection [16]. In contrast, SLY is a full-length 54 kDa thiol-activated secreted hemolysin that has been demonstrated to play an important role in the pathogenesis of *S. suis* infection and inflammatory response in vitro and in vivo [18,50]. The differences between these two *S. suis* antigens in protective efficacy, antibody-mediated phagocytic activities, as well as in their induction of IFN-γ may be attributable to functional differences between the proteins or differences in their subcellular distribution or expression [51]. How these multiple parameters influence immunogenicity/efficacy will be the subject of further investigation.

In conclusion, both GMD and SLY were delivered by the BoHV-4 vectors in an antigenic form that was recognized by sera from SS2-infected rabbits. BoHV-4/GMD and BoHV-4/SLY heightened the production of both IFN-γ and IL-4 and induced antibody response against SS2, SS7, and SS9 in rabbits. Notably, vaccination of rabbits with BoHV-4/GMD provided a high and significant level of protection against SS2 challenge compared to BoHV-4/SLY. In addition, sera from animals vaccinated with BoHV-4/GMD, but not BoHV-4/SLY, promoted a significant level of phagocytic activity by PAMs against SS2, SS7, and SS9. These data suggest that BoHV-4/GMD is a promising vaccine candidate against *S. suis* disease and warrants further validation in a pig model of *S. suis* disease.

## Data Availability

Not applicable.

## Supplementary Materials

The following supporting information can be downloaded at: https://www.mdpi.com/article/10.3390/vaccines11051004/s1, Figures S1 and S2: Stability of *S. suis* transgene expression.
