# Supplementary material for: Bovine Herpesvirus-4 Based Vaccine Provides Protective Immunity against Streptococcus suis Disease in a Rabbit Model
Source: Vaccines (Basel). 2023 May 20;11(5):1004. doi: 10.3390/vaccines11051004 (PMC10222682; doi:10.3390/vaccines11051004)
Supplement: Supplementary file 1 [file vaccines-11-01004-s001.zip › Supplementary Figure S1.pptx]

## Slide 1
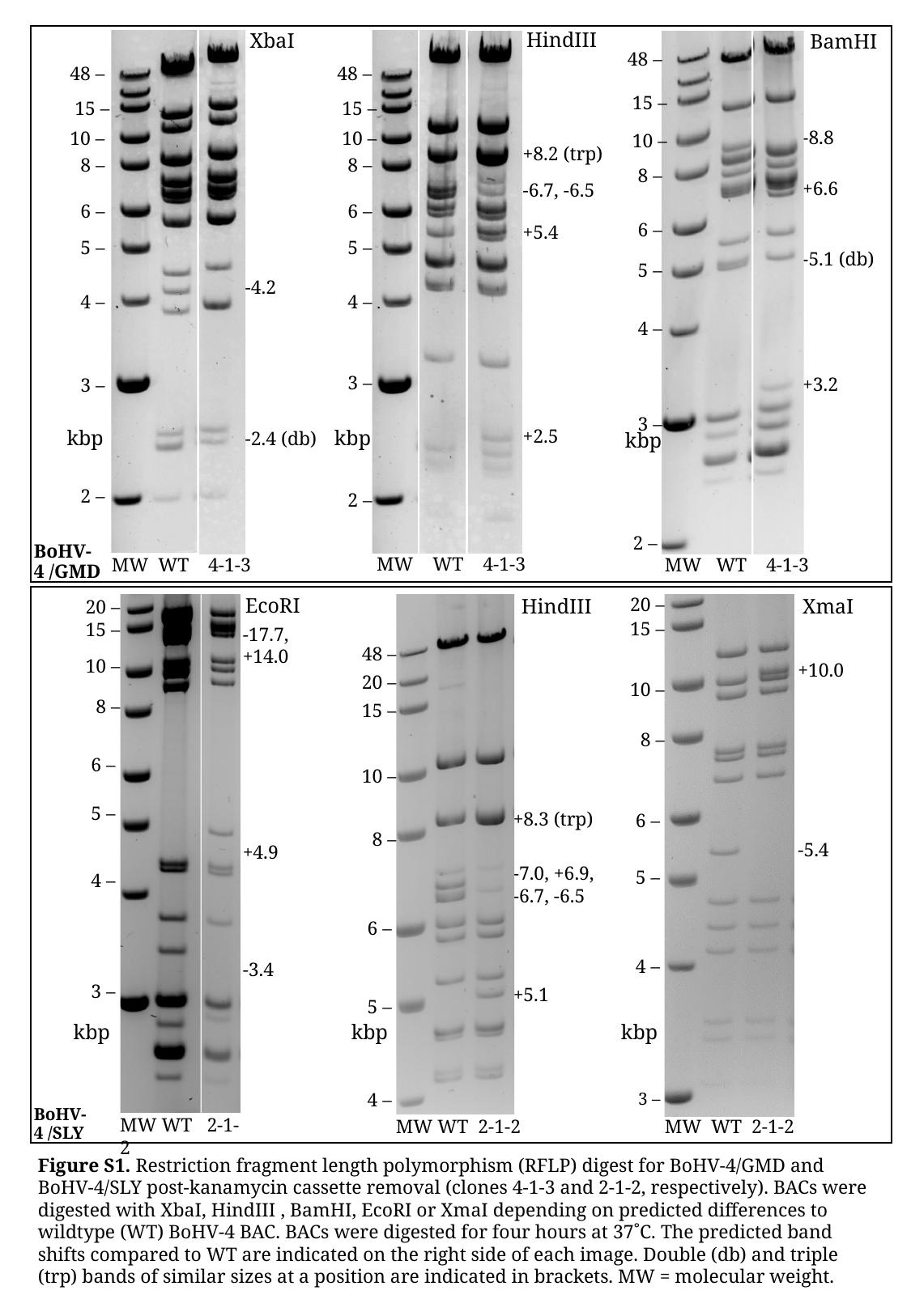

HindIII
XbaI
BamHI
48 –
15 –
10 –
8 –
6 –
5 –
4 –
3 –
2 –
48 –
15 –
10 –
8 –
6 –
5 –
4 –
3 –
2 –
48 –
15 –
10 –
8 –
6 –
5 –
4 –
3 –
2 –
-8.8
+6.6
-5.1 (db)
+3.2
+8.2 (trp)
-6.7, -6.5
+5.4
+2.5
-4.2
-2.4 (db)
kbp
kbp
kbp
BoHV-4 /GMD
MW WT 4-1-3
 MW WT 4-1-3
MW WT 4-1-3
20 –
15 –
10 –
8 –
6 –
5 –
4 –
3 –
EcoRI
HindIII
XmaI
20 –
15 –
10 –
8 –
6 –
5 –
4 –
3 –
-17.7, +14.0
+4.9
-3.4
48 –
20 –
15 –
10 –
8 –
6 –
5 –
4 –
+10.0
-5.4
+8.3 (trp)
-7.0, +6.9, -6.7, -6.5
+5.1
kbp
kbp
kbp
BoHV-4 /SLY
MW WT 2-1-2
 MW WT 2-1-2
MW WT 2-1-2
Figure S1. Restriction fragment length polymorphism (RFLP) digest for BoHV-4/GMD and BoHV-4/SLY post-kanamycin cassette removal (clones 4-1-3 and 2-1-2, respectively). BACs were digested with XbaI, HindIII , BamHI, EcoRI or XmaI depending on predicted differences to wildtype (WT) BoHV-4 BAC. BACs were digested for four hours at 37˚C. The predicted band shifts compared to WT are indicated on the right side of each image. Double (db) and triple (trp) bands of similar sizes at a position are indicated in brackets. MW = molecular weight.
