# Supplementary material for: Bovine Herpesvirus-4 Based Vaccine Provides Protective Immunity against Streptococcus suis Disease in a Rabbit Model
Source: Vaccines (Basel). 2023 May 20;11(5):1004. doi: 10.3390/vaccines11051004 (PMC10222682; doi:10.3390/vaccines11051004)
Supplement: Supplementary file 1 [file vaccines-11-01004-s001.zip › Supplementary Figure S2.pptx]

## Slide 1
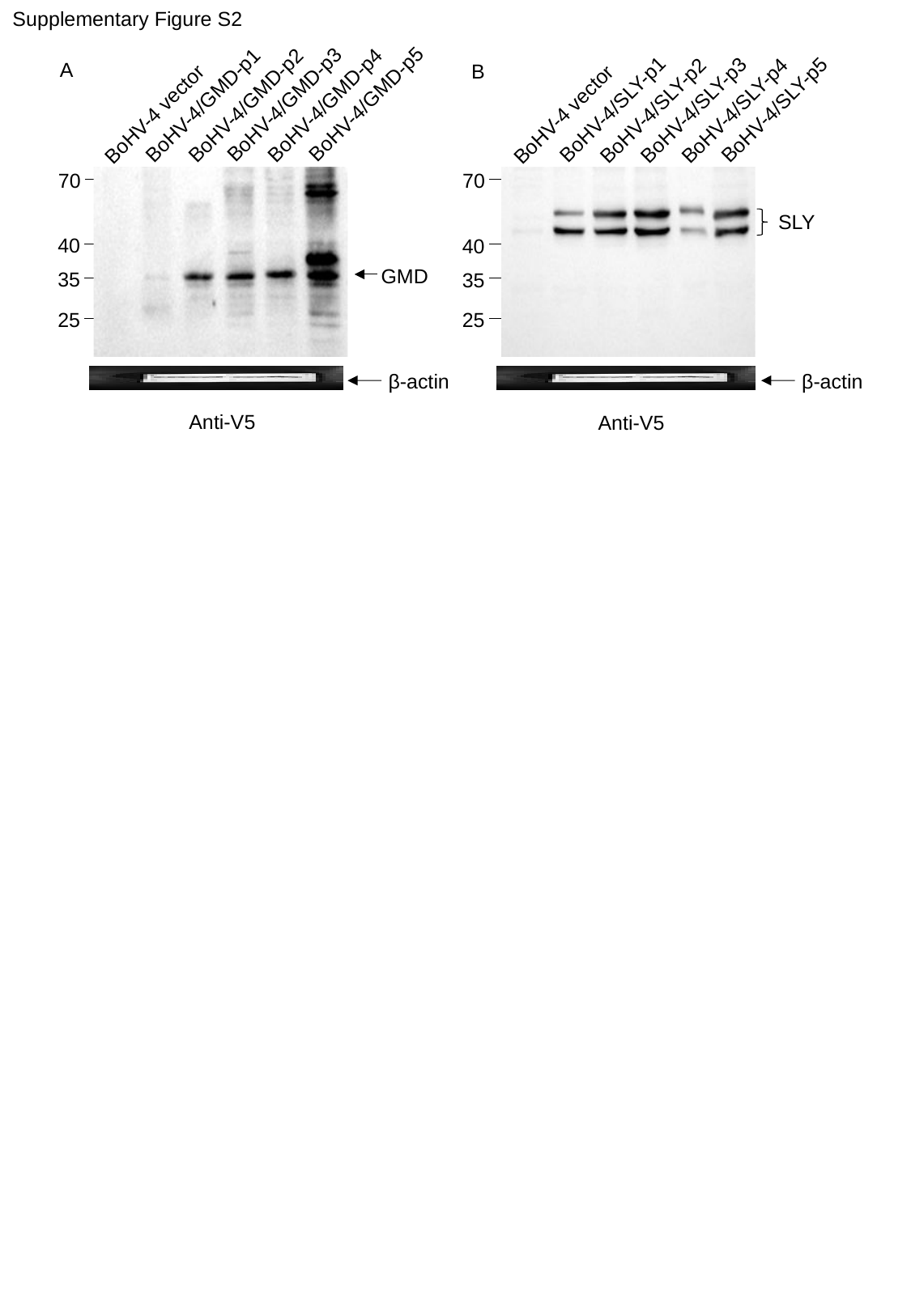

Supplementary Figure S2
A
B
BoHV-4/GMD-p5
BoHV-4/GMD-p3
BoHV-4/GMD-p1
BoHV-4/GMD-p4
BoHV-4/GMD-p2
BoHV-4 vector
70
40
35
25
GMD
BoHV-4/SLY-p1
BoHV-4/SLY-p5
BoHV-4/SLY-p3
BoHV-4/SLY-p4
BoHV-4/SLY-p2
BoHV-4 vector
70
40
35
25
SLY
Anti-V5
Anti-V5
β-actin
β-actin
